# Lessons Learned from COVID-19 Pandemic in Combating Antimicrobial Resistance—Experience of Hong Kong, China

**DOI:** 10.3390/microorganisms12122635

**Published:** 2024-12-19

**Authors:** Edmond Siu-Keung Ma, Shuk-Ching Wong, Vincent Chi-Chung Cheng, Hong Chen, Peng Wu

**Affiliations:** 1 Infection Control Branch, Centre for Health Protection, Department of Health, Hong Kong, China; chenhong@dh.gov.hk; 2 Infection Control Team, Queen Mary Hospital, Hong Kong West Cluster, Hong Kong, China; shchwong@hku.hk (S.-C.W.); vcccheng@hku.hk (V.C.-C.C.); 3 Department of Microbiology, School of Clinical Medicine, Li Ka Shing Faculty of Medicine, The University of Hong Kong, Hong Kong, China; 4 Department of Microbiology, Queen Mary Hospital, Hong Kong, China; 5 World Health Organization Collaborating Centre for Infectious Disease Epidemiology and Control, School of Public Health, Li Ka Shing Faculty of Medicine, The University of Hong Kong, Hong Kong, China; pengwu@hku.hk; 6 Laboratory of Data Discovery for Health (D24H), Hong Kong Science and Technology Park, Hong Kong, China

**Keywords:** antimicrobial resistance, COVID-19, pandemic, public health

## Abstract

The world has gone through the COVID-19 pandemic and has now returned to normalcy. We reviewed the strategies and public health actions conducted in Hong Kong during the COVID-19 pandemic, and reflected on the lessons learned, which are potentially useful in the fight against antimicrobial resistance (AMR). We recommended extending wastewater surveillance for AMR, apart from SARS-CoV2. We suggested exploring the use of rapid tests in outpatients to aid clinical diagnosis and reduce antibiotic use for viral infections. Stringent infection control measures are crucial to prevent nosocomial transmission of resistant microorganisms, such as vancomycin-resistant enterococci and carbapenemase-producing *Enterobacterales* in hospitals and in elderly homes. Taking COVID-19 experiences as a reference, transparent data, the prompt dissemination of information, and strategic risk communication should be adopted to maintain sustained behavioral changes in AMR. We also encouraged the adoption of information technology, artificial intelligence, and machine learning in antimicrobial stewardship programs. We also discussed the potential merits and limitations of these strategies. The lessons learned from the COVID-19 pandemic may provide insights into the long battle against AMR.

## 1. Introduction

The world has gone through the COVID-19 pandemic and has now returned to a state of normalcy. However, the global problem of antimicrobial resistance (AMR) has been silently ongoing and has worsened in some regions over the past few years [1,2,3]. Studies have reported the extensive use of antimicrobials, including amoxicillin/clavulanic acid, ceftriaxone, piperacillin/tazobactam, and levofloxacin, for COVID-19 patients, not only in the early phase of the pandemic, but also when the highly transmissible omicron variant spread globally [4,5]. The Centers for Disease Control and Prevention in the United States reported an alarming increase in infections with antimicrobial resistant organisms during hospitalizations, with rates increasing by over 15% from 2019 to 2020 [2]. Reports from other parts of the world also indicated an increase in the resistance of methicillin-resistant *Staphylococcus aureus*, vancomycin-resistant enterococci, carbapenem-resistant *Acinetobacter baumannii*, and carbapenem-resistant *Enterobacterales* during the COVID-19 pandemic [1,6]. In a recent study on the global burden of AMR, it was estimated that 4.71 million deaths were directly associated with bacterial AMR in 2021, including 1.14 million deaths attributable to bacterial AMR [7]. The study predicts that an estimated 1.91 million deaths attributable to AMR and 8.22 million deaths associated with AMR could occur globally in 2050.

The 79th United Nations General Assembly convened a high-level meeting on AMR in September 2024, after the first meeting held in 2016. This meeting encouraged world leaders to collectively address the looming threat of AMR to global health, using a One-Health approach, leading to a commitment to a 10% reduction in the annual estimated 4.95 million deaths associated with bacterial AMR by 2030. It is time to regain focus and resources to combat this AMR pandemic, and we provide lessons learned from the battle against COVID-19, which can serve as useful strategies for this purpose [8]. The potential merits and limitations of these strategies are also discussed.

## 2. Novel Sewage Surveillance to Track the Trends of AMR

Polluted soils and water bodies are potential reservoirs where antibiotic-resistant bacteria can spread the resistant genes between species, subsequently entering the transmission chain from food to animals to humans. The United Nations Environmental Programme highlighted the importance of environmental surveillance for AMR in its report in 2022. The COVID-19 pandemic has reactivated the momentum to adopt wastewater surveillance as a resource-efficient tool for monitoring the activities of SARS-CoV-2. Sewage surveillance in Hong Kong has been demonstrated to detect silent COVID-19 transmission and facilitate real-time intervention [9]. This novel surveillance strategy has provided an impetus for AMR monitoring in the population and assessment of environmental transmission risks, augmenting traditional surveillance that relies on clinical laboratory data from human samples. This strategy has been implemented in the United Kingdom, Switzerland, and India [10,11,12]. Some studies have suggested promising results regarding the high concordance between wastewater and human samples when estimating AMR prevalence [13]. Sewage surveillance has the advantages of high spatial coverage with large communities, and hence a low testing cost per capita. For example, the national sewage monitoring program in the Netherlands consisted of about 300 municipal wastewater treatment plants, which could cover 99.6% of the 17 million people in the country [14]. In the United States, wastewater treatment plants collect sewage from nearly 80% of households, and the sewer network ranges in size from less than 2000 to >3 million persons [15]. It can help identify hotspots, such as pharmaceuticals, hospitals, and animal farms, which contribute to the spread of clinically relevant AMR [16]. In addition, it can bridge the missing epidemiological links between humans, animals, and the environment in the evolution of AMR. The Tricycle Protocol for enumerating extended-spectrum beta lactamase-producing *Escherichia coli*, launched by the World Health Organization (WHO), exemplifies such initiatives across the One-Health spectrum [17].

Samples taken from the influents of water treatment plants may reflect potential sources of AMR, whereas samples taken from effluents can assist in determining environmental transmission risks [18]. Water samples can be tested for antibiotic residues, resistant bacteria, and resistance genes [18]. Standardized laboratory methods and reporting endpoints are essential for the meaningful comparison of data to inform policies and actions [19]. The Global Sewage Surveillance Consortium has attempted to identify regional patterns, diverse genetic environments of common antimicrobial resistance genes, and specific transmission patterns using metagenomic sequencing [20]. Further research is encouraged to monitor the temporal trends of AMR and to detect any changes potentially associated with interventions and public health actions. However, it should be noted that unlike isolate-based surveillance, sewage-based metagenomic surveillance does not link AMR genes to specific bacterial species. Because the origins of sewage bacteria are often unknown, interpreting sewage surveillance data can be challenging.

## 3. Use of Rapid Tests to Assist Clinical Diagnosis and Reduce Antibiotic Use

During the COVID-19 pandemic, similar to in many other areas, the community in Hong Kong was well adapted to the use of inexpensive rapid antigen tests for self-diagnosis of SARS-CoV-2 infections. The use of these rapid tests can potentially reduce the overuse of antibiotics for treating viral diseases, especially during influenza season when disease prevalence is high, thus increasing the positive predictive value of the test. C-reactive protein point-of-care tests have been shown to reduce antibiotic prescriptions in primary care patients presenting with symptoms of acute respiratory infection [21]. In a meta-analysis of the efficacy of rapid tests to guide antibiotic prescriptions for a sore throat in primary care settings, results from five trials involving 2545 participants showed an overall 25% reduction in prescribed antibiotics among the group receiving rapid tests, compared with management based on clinical assessment [22]. With commercial diagnostic kits now becoming more readily available for testing multiple pathogens, including SARS-CoV-2, influenza A and B, parainfluenza, respiratory syncytial virus, adenovirus, and mycoplasma pneumoniae, we call for further research to investigate the effectiveness of these diagnostics in reducing antibiotic consumption. In addition to antibiotic use, other endpoints, including clinical outcomes of patients, number of follow-up consultations, willingness of physicians, and acceptability of patients to undergo the rapid tests, should also be evaluated. Although the use of antibiotics to treat viral diseases is not recommended, due to the possibility of causing more bacterial resistance in the future, antibiotics should be administered when the morbidity involves a comorbidity of bacterial infection.

## 4. Stringent Infection Control Practices to Reduce Transmission Risk in Hospitals and Institutions

The importance of infection control to combat the transmission of COVID-19 is beyond doubt. Healthcare professionals have been intensively trained to comply with stringent infection control practices to prevent nosocomial infections within hospitals and other isolation facilities [23,24]. The use of personal protective equipment, including masks, gloves, and gowns, with a risk-based approach and proper donning and doffing techniques, have been integrated as standard procedures of patient management. This applies not only to patients with respiratory infections but also to those infected with multi-drug-resistant organisms, such as vancomycin-resistant Enterococci and carbapenemase-producing Enterobacteriaceae, in both hospitals and elderly homes in Hong Kong [25,26]. Ultraviolet-C has been increasingly utilized for environmental disinfection during the COVID-19 pandemic [27,28]. It can be considered a valuable tool in managing hospital outbreaks by reducing transmission through surfaces contaminated with resistant organisms like *Acinetobacter baumannii*, which can survive for prolonged periods in the environment [29,30]. Similarly, air purifiers were commonly used to improve ventilation during the pandemic in hospitals, quarantine centers, and elderly homes in Hong Kong. This approach may be useful for reducing the spread of other multi-drug-resistant organisms, such as *Candida auris*, which pose a transmission risk through air dispersal [31,32].

## 5. Strategic Risk Communication to Drive Sustainable Behavioral Changes in the Community

Non-pharmacological interventions, including mask wearing, hand hygiene, social distancing, and environmental disinfection, contributed to containing the disease in the early phase of the COVID-19 pandemic, when vaccines and antivirals had not yet been developed. Evidence has shown that these interventions also successfully reduced respiratory infections other than COVID-19 [33]. In Hong Kong, the sustained implementation of public health interventions has proven effective against respiratory microbes such as scarlet fever, tuberculosis, and chickenpox, coupled with a reduction in the overall antibiotic consumption [34]. Transparent data, the prompt dissemination of information, and strategic risk communication are key drivers in changing people’s risk perceptions and promoting the adoption of hygiene practices and health actions, as demonstrated by the increasing uptake of COVID-19 vaccines. Further health promotion activities regarding AMR can take reference from experiences gained during the COVID-19 pandemic. For instance, the public should be aware that similarly to SARS-CoV-2, resistant bacteria can be transmitted from person to person and may lead to serious clinical outcomes, including death if infected. As advocated by the WHO, AMR is invisible, but its victims are not [35]. Publicity campaigns can be conducted by having patients that have survived serious AMR infections to share real-life experiences, to illustrate its tangible health impact.

## 6. Use of Information Technology to Improve Efficiency of Clinical Management and Public Health Interventions

The COVID-19 pandemic has significantly accelerated the use of telehealth and innovative information technology to support patient management and public health functions [36]. A wide array of electronic platforms has been developed in Hong Kong to assist port health control, epidemiological investigations, contact tracing, quarantine, isolation, disease surveillance, laboratory testing, law enforcement, and risk communication [37]. The rapid development of artificial intelligence, particularly its application in healthcare, has provided opportunities to identify resistant bacteria more efficiently. Strengthening antimicrobial stewardship programs in public hospitals using information technology has been identified as one of the priority interventions in the latest Hong Kong Strategic and Action Plan on AMR [38]. A systematic review including 18 studies conducted in the United States, Italy, Israel, the Netherlands, Greece, and Taiwan concluded that machine learning algorithms were useful to assist antimicrobial stewardship teams in multiple tasks, such as identifying inappropriate prescribing practices, choosing appropriate antibiotic therapy, and predicting AMR [39]. A pilot program has been initiated to generate automated reminders to alert physicians to review the appropriateness of prescribing broad-spectrum antibiotics, thereby saving time in analyzing antibiotic prescriptions. We are also developing a user-friendly mobile application to promote guideline-conformed antibiotic prescriptions in hospitals and using social media platforms to improve publicity campaigns [40]. In Hong Kong, deep neural networks have been shown to be superior to logistic regression in predicting ESBL production in *Enterobacterales* causing community-onset bacteremia, which offers clinical utility in guiding judicious empirical antibiotic use [41]. In other areas, a combination of machine learning algorithms and laboratory testing has been suggested to accelerate the process of discovering new antimicrobials [42].

## 7. Conclusions

With the global concerns regarding emerging resistant pathogens, novel strategies are needed to tackle this public health problem. Taking reference from what we have learned from the COVID-19 pandemic, we suggest adopting strategic measures to combat AMR, including the utilization of wastewater for AMR surveillance, the employment of rapid tests to aid clinical diagnosis and reduce unnecessary antibiotic use, the implementation of stringent infection control measures in healthcare settings, sustained health promotion through strategic risk communication, and the application of information technology such as artificial intelligence and machine learning to improve the efficiency of clinical management and public health interventions. In addition, we advocate for conducting further studies to examine the feasibility and real-life impact of these new approaches in control of AMR.

## Data Availability

No new data were created or analyzed in this study.
